# Effects of supplementation of green tea extract on the milk performance of peripartal dairy cows and the expression of stress response genes in the liver

**DOI:** 10.1186/s40104-020-00465-y

**Published:** 2020-06-05

**Authors:** Denise K. Gessner, Corinna Brock, Lena M. Hof, Erika Most, Christian Koch, Klaus Eder

**Affiliations:** 1 Institute of Animal Nutrition and Nutrition Physiology, Justus-Liebig-University Giessen, Heinrich-Buff-Ring 26-32, 35392 Giessen, Germany; 2 Educational and Research Centre for Animal Husbandry, Hofgut Neumühle, 67728 Münchweiler an der Alsenz, Germany

**Keywords:** Animal nutrition, Cow, Green tea extract, Liver, Metabolism, Milk performance

## Abstract

**Background:**

We hypothesised that supplementation of green tea extract (GTE) in dairy cows during the transition period can attenuate proinflammatory conditions and prevent endoplasmic reticulum (ER) stress in the liver of these cows. Thirty Holstein cows with an average parity of 3.06 (± 1.31, SD) were divided into a control group and a group that received a daily amount of 10 g of GTE from d 7 before the calving day and a daily amount of 20 g of GTE from the day of calving until d 7 of lactation.

**Results:**

Cows supplemented with GTE did not show differences in energy intake or milk yield in weeks 2–7 of lactation. However, these cows had a lower milk fat concentration and a lower energy corrected milk yield than the control cows and showed a trend of improved energy balance. The relative mRNA concentrations of proinflammatory genes, genes involved in the acute phase reaction and antioxidant genes in the liver in weeks 1, 4 and 7 of lactation were not different between the two groups of cows. The concentrations of α-tocopherol and the Trolox equivalent antioxidant capacity in plasma were not different between the two groups. However, the group supplemented with GTE showed significant reductions of some genes of the unfolded protein response (UPR) in week 1 and a trend of lower liver triacylglycerol (TAG) concentrations in the liver compared to the control group.

**Conclusions:**

This study shows that supplementation of GTE in dairy cows lowers the fat concentration in the milk but overall has no effect on the expression of inflammatory genes and the antioxidative status in dairy cows during early lactation. The finding of reduced mRNA levels of genes involved in the UPR at week 1, however, supports other results showing that supplementation of polyphenols could prevent the development of ER stress in the liver of cows during early lactation. The finding of a tendency towards a reduced TAG concentration in the liver of cows supplemented with GTE might be due to an improved energy balance in these cows.

## Background

During the transition period, spanning the time 3 weeks before to 3 weeks after parturition, the liver of dairy cows tends to develop a proinflammatory status [1–3]. This proinflammatory condition is caused by infectious diseases, such as mastitis, metritis, leaky gut syndrome or several stressors, such as social stress or heat stress [4]. The development of an inflammatory condition in the liver not only requires energy for the activation of the immune system and amino acids for the production of acute phase proteins but also leads to an impairment of hepatic metabolism [4, 5]. During proinflammatory conditions, the production of acute phase proteins is enhanced at the expense of negative acute phase proteins, such as proteins involved in normal metabolism, including enzymes involved in β-oxidation or proteins required for the secretion of triacylglycerols (TAG) from the liver into the blood [6, 7]. It has been shown that inhibition of the proinflammatory condition by administration of anti-inflammatory drugs during the first days of lactation not only improves liver function but also increases milk yield and lowers somatic cell counts in the milk, indicative of improved health of the mammary gland [8, 9].

Recent studies have found that supplementation of dairy cows with plants rich in polyphenols, such as a grape seed and grape marc meal extract or an extract from *Scutellaria baicalensis,* can increase the milk yield in dairy cows [10, 11]. It was also observed that supplementation of grape seed and grape marc meal extract in dairy cows attenuated the development of endoplasmic reticulum stress (ER) stress, another phenomenon that occurs in dairy cows in early lactation [12]. Polyphenols exert anti-inflammatory effects, and the beneficial effects of plants rich in polyphenols observed in dairy cows might be, at least in part, due to inhibition of the inflammatory process occurring during the transition period [10, 12].

Green tea is another source rich in polyphenols [13]. Several studies in rodents have shown that polyphenols from green tea exert strong anti-inflammatory effects in various tissues, including the liver [14, 15]. The present study investigated the hypothesis that supplementation of green tea in dairy cows during the transition period can attenuate the proinflammatory condition and prevent ER stress and in turn has beneficial effects on lipid metabolism and milk performance in dairy cows.

## Methods

### Animals and diets

This study was carried out at the Educational and Research Centre for Animal Husbandry Hofgut Neumühle (Münchweiler and der Alsenz, Germany). The trial included 30 multiparous Holstein cows with an average parity number of 3.06 (± 1.31, SD). The animals were allocated to two groups, a control group [*n* = 15, average parity number: 2.80 (± 1.01, SD)] or a group supplemented with a product consisting of green tea extract [GTE, n = 15, average parity number: 3.33, (± 1.54, SD)]. The number of cows per group was determined by performing a biometric analysis with G*Power (rel. 3.1.2) to detect a significant difference between the groups (main parameter: energy-corrected milk) assuming a hypothesized effect size of 1.10, a type I error of 0.05 (two-sided) and a statistical power of 0.80. The hypothesized size effect was calculated based on the effect (means and SD) of green tea extract and curcuma on energy-corrected milk output in dairy cows [16]. Allocation of the cows into the two groups was performed according to their previous performance parameters (data of the previous 305 d lactation period, control group vs. GTE group: milk yield, 10,153 vs. 10,788 kg; milk fat concentration, 3.86 vs. 3.73%; milk protein concentration, 3.21% vs. 3.18%; milk fat, 392 vs. 402 kg; milk protein, 326 vs. 342 kg). The body weights and the body condition scores of the cows at week 3 ante partum did not differ between the two groups (control group vs. GTE group: body weights, 777 ± 73 vs. 768  ± 79 kg; body condition score, 3.70 ± 0.44 vs. 3.46 ± 0.32; means ± SD). The GTE was supplemented from d 7 before the expected day of calving until d 7 after calving. In the period from d 7 before the expected day of calving, a daily amount of 10 g of GTE per cow was supplemented; in the period between the day after calving and d 7 after calving, a daily amount of 20 g of GTE per cow was supplemented. As some cows did not calve at the expected day of calving, the actual number of days antepartum that the cows received GTE was 7.7 ± 4.3 (mean ± SD, range: 2–16 d). The supplemented product consisted of a spray-dried extract of green tea (Fa. Martin Bauer, Vestenbergsgreuth, Germany). The product had a total polyphenol concentration of 351 mg gallic acid equivalents per g, consisting of 255 mg of tannic polyphenols and 96 mg of nontannic polyphenols (according to determination of total polyphenol concentration by the Folin-Ciocalteu method [17]). The daily amount of the supplement was mixed into 500 g of concentrate, which was administered to the cows by hand after milking. The control group received 500 g of concentrate without the addition of GTE.

The cows were fed two basal total mixed rations (TMR). The TMR fed during the dry period (until the day of calving) was calculated to meet the demand of net energy and crude protein (CP) of a dry cow with a body weight of 650 kg and an assumed dry matter intake (DMI) of 12 kg (Table 1). After calving, the cows were offered a TMR calculated to meet the requirement for energy and CP for a milk yield of 34 kg, with an assumed DMI of 22 kg (Table 1). Five days before the estimated parturition date until 5 d after parturition, the cows were kept separately from the herd in straw-bedded calving pens. The feed intake of the individual cows could not be recorded during this time. After the cows were transferred to the freestall barn, the individual feed intake of the cows was recorded by an electronic feeding system (Roughage Intake Control; Insentec B.V., Marknesse, the Netherlands).

**Table 1:** Composition of the basal total mixed rations used as experimental diets during dry period and lactation

	Dry period (week 3 a.p. – calving)	Lactation (calving – week 7 p.p.)
Component, % of DM		
Corn silage	37.8	18.3
Grass silage	37.7	24.4
Concentrate^1^	14.1	34.6
Hay	6.9	4.1
Straw	2.3	2.8
Pressed sugar beet pulp	–	14.5
Urea	0.5	–
Vitamin and mineral premix^2^	0.8	–
Fat	–	0.9
Sodium hydrogen carbonate	–	0.4
Energy and nutrient contents		
Net energy for lactation, MJ/kg DM	6.4	7.0
Crude fibre, % of DM	20.6	17.5
NDF, % of DM	38.2	33.3
ADF, % of DM	22.3	21.0
Crude protein, % of DM	13.2	17.1
Available crude protein, % of DM^3^	13.8	15.7
Ruminal N balance, g/kg^3,4^	−1.0	2.3

^1^ Concentrate (Raiffeisen Waren-Zentrale Rhein-Main, Köln, Germany) consisted of (% of dry matter): rapeseed meal, 25.0; soybean meal, 22.5; corn, 17.7; barley, 15.8; soybean hulls, 12.2; melasses, 1.7; mineral mix, 1.9; urea, 1.1; sodium chloride, 1.0; fat, 0.8; calcium carbonate, 0.3. The mineral mix provided (per kg concentrate): Sodium, 1.52 g; magnesium, 0.95 g; calcium, 3.42 g; phosphorus, 0.67 g; iron, 0.57 mg; manganese, 95 mg; zinc, 152 mg; copper, 38 mg; iodine 1.9 mg; cobalt, 1.52 mg; selenium, 0.95 mg; vitamin A, 22,800 IU; vitamin D, 2850 IU; vitamin E, 114 mg

^2^ The vitamin and mineral premix (Rindamin K11 ATG; Schaumann, Pinneberg, Germany) provides per kg TMR: Calcium, 0,36 g; phosphorus, 0,36 g; sodium, 0.36 g; magnesium, 0.4 g; zinc, 28 mg; manganese, 17 mg; copper, 6.0 mg; cobalt, 0.24 mg; iodine, 0.8 mg; selenium, 0.21 mg; vitamin A, 4,000 IU; vitamin D, 600 IU; vitamin E, 20 mg

^3^Calculated according to GfE [18]

^4^Ruminal N balance = (crude protein – available protein)/6.25

### Feed samples and analysis

The feed components were sampled fortnightly and stored at − 20 °C for further analysis. Feed samples were analysed for crude ash, CP, ether extract and crude fibre according to the official methods of Verband der Deutschen Landwirtschaftlichen Untersuchungs-und Forschungsanstalten [19]. Concentrations of neutral detergent fibre (NDF) and acid detergent fibre (ADF) were determined according to Van Soest et al. [20]. Net energy lactation (NEL) and CP of the diets were calculated according to Gesellschaft für Ernährungsphysiologie (GfE) [18].

### Collection of milk and blood samples and liver biopsies

Cows were milked twice daily (05:00 h, 15:30 h) in a combined milking parlour offering space for eight cows in the herringbone parlour and a side-by-side parlour for 10 cows (GEA Farm Technologies, Boenen, Germany). The daily milk yield was recorded electronically via the herd management system Dairy Plan C21 from GEA. From week 1 to week 7 postpartum, milk aliquots from one evening and the next morning were taken once per week and pooled for further analysis of milk fat, protein and lactose by infrared spectrophotometry using a MilkoScan FT6000 (Foss Analytical A/S, Hillerød, Denmark). Energy-corrected milk (ECM), adjusted to 4% fat and 3.4% protein content, was calculated according to GfE [18].

Blood samples were collected from all cows in the morning before feeding at week 1 (d 6–12), week 4 (d 26–32) and week 7 (d 49–53) of lactation. The blood samples were taken via puncture of the coccygeal vessels into tubes containing ethylenediaminetetraacetic acid (S-Monovette®, Sarstedt, Nümbrecht, Germany) and were immediately centrifuged. Plasma, obtained by centrifugation of blood at 3,500×*g* for 15 min, was frozen (− 20 °C) for further analysis. In addition, liver biopsies were taken directly after blood sampling as described previously [10].

### Analysis of plasma and liver samples

Concentrations of nonesterified fatty acids (NEFA), β-hydroxybutyrate (BHBA), TAG, total cholesterol and albumin in the plasma were determined by enzymatic reagent kits, following the instructions of the manufacturer (Codes No: 436–91995 and 417–73501, Wako Chemicals GmbH, Neuss, Germany; Fluitest® TG, Fluitest® ALB, Fluitest® Chol, Analyticon, Lichtenfels, Germany). The concentrations of serum amyloid A (SAA), haptoglobin (HP) and retinol binding protein 4 (RBP4) in the plasma were determined by commercial enzyme-linked immunosorbent assay kits (EB0015, EB0011, EB0929b, Hölzel, Cologne, Germany). The Trolox equivalent antioxidative capacity (TEAC) was measured following the protocol of Re et al. [21]. The concentrations of α-tocopherol and β-carotene in the plasma were determined by high-performance liquid chromatography (L-7100, LaChrom, Merck-Hitachi, Darmstadt, Germany) with slight modifications of the method of Balz et al. [22] as described in detail by Gessner et al. [19]. For the determination of lipid concentrations in the liver, small samples of the liver biopsies were extracted with a mixture of hexane and isopropanol (3:2, v/v, according to [23]). The solvent of an aliquot of the lipid extract was evaporated by vacuum, and the dried lipids were dissolved in Triton X-100. The concentrations of TAG and cholesterol were determined by adding enzymatic kit reagents (Fluitest® TG, Fluitest® CHOL, Analyticon) to the dissolved lipids [24]. Quality control of all the methods used was performed by repeated measurements of a pooled cow plasma sample. For TAG and cholesterol determinations, control plasma samples with known concentrations supplied by the manufacturer were determined for quality control of the assays. Intraday (*n* = 3–5) and interday (*n* = 2–5) coefficients of variation of the analyses were as follows: NEFA, 2.5% and 5.2%; BHBA, 2.9% and 1.8%; TAG in plasma, 3.2% and 4.7%; TAG in liver, 3.3% and 8.7%; cholesterol in plasma, 4.5% and 5.6%; cholesterol in liver, 6.2% and 11.3%; albumin, 2.4% and 3.1%; SAA, 9.6% and 4.8%; HP, 9.9% and 7.5%; RBP4, 7.1% and 5.6%; TEAC, 2.6% and 4.1%; α-tocopherol, 2.8% and 4.7%; β-carotene, 4.4 and 3.8%.

### Real-time quantitative polymerase chain reaction (qPCR)

Relative mRNA abundancies of the following genes were determined by qPCR: Acetyl-CoA carboxylase (*ACACA*)*,* acyl-CoA dehydrogenase medium chain (*ACADM*)*,* acetyl-CoA acetyl transferase 1 (*ACAT1*), acyl-CoA oxidase 1 (*ACOX1*); apolipoprotein B (*APOB*), activating transcription factor 4 (*ATF4*); BCL2-antagonist/killer 1 (*BAK1*), BCL2 associated X, apoptosis regulator (*BAX*), caspase 3 (*CASP3*), caspase 8 (*CASP8*), catalase (*CAT*), C-C motif chemokine ligand 2 (*CCL2*), ceruloplasmin (*CP*), carnitine palmitoyl transferase 1A (*CPT1A*), C-reactive protein (*CRP*), interleukin 8 (*CXCL8*), DNA-damage-inducible transcript 3 (*DDIT3*), DnaJ heat shock protein family (Hsp40) member C3 (*DNAJC3*), ER degradation enhancing alpha-mannosidase like protein 1 (*EDEM1*), fatty acid synthase (*FASN*), glutathione peroxidase 1 (*GPX1*), homocysteine inducible ER protein with ubiquitin like domain 1 (*HERPUD1*), haptoglobin (*HP*), interleukin 1 beta (*IL1B*), 3-hydroxymethyl-3-methylglutaryl-CoA lyase (*HMGCL*), 3-hydroxy-3-methylglutaryl-CoA synthase 2 (*HMGCS2*), heat shock protein family A (Hsp70) member 5 (*HSPA5*), metallothionein-1A (*MT1A*), microsomal triglyceride transfer protein (*MTTP*), NAD(P)H-quinon oxidoreductase (*NQO1*), protein disulfide isomerase family A member 4 (*PDIA4*), prostaglandin endoperoxide synthase 2 (*PTGS2*), retinol-binding Protein 4 (*RBP4*), serum amyloid A3 (*SAA3*), superoxide dismutase 1 (*SOD1*), sterol regulatory element-binding transcription factor 1 (*SREBF1*), tumor necrosis factor (*TNF*), UDP-glucuronosyl transferase family 1 member A1 (*UGT1A1*), spliced X-box binding protein 1 (*XBP1s*). The isolation of total ribonucleic acid (RNA) from liver biopsies, cDNA synthesis, qPCR, and calculations were carried out as described by Gessner et al. [25, 26]. According to Vandesompele et al. [27], the geNorm normalization factor was used to investigate the expression values of the genes, with eukaryotic translation elongation factor 1 alpha 1 (*EEF1A1*), H3.3 histone A (*H3F3A*), and ribosomal protein L12 (*RPL12*) being the 3 most stable of 8 tested candidate reference genes in the liver. Gene-specific primer pairs were designed using Primer3 and BLAST. Most primer pairs were designed to span an exon-exon junction. The primers were synthesized by Eurofins MWG Operon (Ebersberg, Germany). Details about the gene-specific primer pairs for the genes considered in this study are shown in Additional file 1: Table S1. PCR amplification products were verified using 1.5% agarose gel electrophoresis, stained with GelRedTM nucleic acid gel stain (Biotium, Hayward, CA, USA) and visualized under UV light with a digital camera (SynGene, Cambridge, England).

### Statistical analysis

Statistical analyses were performed with the statistical software R, version 3.4.2 (R core Team 2017). The data were analysed with a linear mixed-effects model using the packages nlme, lsmeans, multcomp, lattice und predictmeans. The model included treatment, week, parity (2–3 or > 3), and the treatment × week interaction as fixed factors and cow as a random factor to account for repeated measures over the different time points of sampling on the same animal. Residuals were assessed for distribution of normality by Anderson-Darling test and for homoscedasticity by Bartlett tests. Pairwise comparisons between the overall treatment means and the means at each week were performed using linear contrasts for least square means. The level of statistical significance was set at *P* <  0.05.

## Results

### Feed intake, energy balance, milk production and composition

As expected, the week of lactation had significant effects on feed intake, energy balance, milk yield and composition (concentrations of fat, protein and lactose) (Table 2). Feed intake and milk yield were not influenced by GTE supplementation in week 2 to week 7 of lactation. However, the concentrations of fat and lactose in the milk were reduced by GTE supplementation, while the protein concentration remained unchanged. For fat concentration, there was also an interaction between GTE supplementation and the week of lactation. The fat concentration in the milk was significantly reduced in the cows supplemented with GTE in weeks 2, 3, 5 and 6 compared to that in the control cows, whereas there was no difference in the milk fat concentration between the two groups in weeks 4 and 7 (Table 3). For the protein concentration in the milk and the daily amount of protein, no main effects of GTE supplementation were detected; however, significant interactions between GTE supplementation and the week of lactation were observed (Table 2). The daily amount of protein was significantly lower in the GTE-supplemented cows than in the control cows at weeks 5 and 7; in the other wks, there were no differences in the daily amounts of protein between the two groups (Table 3). The protein concentration of the milk did not differ between the two groups of cows at all the weeks considered. As a result of the reduced fat concentration in the milk, the GTE-supplemented group showed a reduced daily amount of fat and a reduced amount of ECM compared the control group (Table 2). The feed efficiency [kg ECM/kg dry matter intake (DMI)] was also reduced in the GTE-supplemented group compared to the control group. Moreover, as a result of the lower ECM, the GTE-supplemented cows tended to show a lower negative energy balance than the control cows.

**Table 2:** Feed intake, milk production, and milk composition of cows fed the control diet or the control diet supplemented with green tea extract (GTE) in average over weeks 2 to 7 of lactation^1^

	Week 2 to week 7			*P*-value			
Variable	Control	GTE	SEM	GTE	Week	GTE × week	Parity
DMI, kg/d	20.2	19.8	0.65	0.67	< 0.001	0.06	0.45
Net energy intake, MJ/d	141	138	4.5	0.67	< 0.001	0.06	0.45
Energy balance, MJ NEL/d	- 45.1	- 35.0	7.0	0.06	< 0.001	0.72	0.44
Milk yield, kg/d	46.1	43.9	1.50	0.17	< 0.001	0.21	0.70
ECM^2^, kg/d	46.6^a^	40.7^b^	1.68	0.002	0.08	0.91	0.53
Feed efficiency, kg ECM/kg DMI	2.36^a^	2.11^b^	0.12	0.010	< 0.001	0.09	0.47
Fat, %	4.29^a^	3.68^b^	0.18	< 0.001	< 0.001	0.003	0.89
Protein, %	3.05	3.08	0.07	0.92	< 0.001	0.02	0.54
Lactose, %	4.83^a^	4.75^b^	0.03	0.026	0.015	0.34	0.44
Fat, kg/d	1.96^a^	1.58^b^	0.10	< 0.001	0.07	0.13	0.74
Protein, kg/d	1.40	1.34	0.05	0.16	0.36	0.031	0.33

^1^Values are least square means, *n* = 15 for each group

^2^ECM, energy corrected milk, adjusted to 4% fat and 3.4% protein

^a,b^ Means with different superscripts differ significantly (*P* < 0.05)

DMI, dry matter intake; SEM, standard error of means

**Table 3:** Concentrations of fat and protein in the milk and daily amount of milk protein of cows fed the control diet or the diet supplemented with green tea extract (GTE) in weeks 2 to 7 of lactation^1^

Week	Fat, %			Protein, %			Protein, kg/d		
	Control	GTE	SEM	Control	GTE	SEM	Control	GTE	SEM
2	4.97^a^	3.57^b^	0.25	3.34	3.53	0.09	1.37	1.39	0.06
3	4.69^a^	4.06^b^	0.26	3.16	3.14	0.09	1.38	1.33	0.06
4	4.34	3.90	0.26	3.01	2.97	0.09	1.39	1.28	0.06
5	4.14^a^	3.54^b^	0.26	2.93	2.80	0.09	1.41^a^	1.26^b^	0.06
6	3.85^a^	3.29^b^	0.25	2.86	2.91	0.09	1.39	1.31	0.06
7	3.71	3.47	0.26	2.91	2.85	0.09	1.41^a^	1.28^b^	0.06

^1^Values are least square means, *n* = 15 for each group

^a,b^ Means within one week with different superscripts differ significantly (*P* < 0.05)

*SEM* standard error of means

### Metabolic and antioxidant parameters in the plasma

Concentrations of NEFA and BHBA were influenced by the week of lactation but were not influenced by GTE supplementation (Table 4). Concentrations of TAG and cholesterol in the plasma were influenced by the week of lactation and by GTE supplementation. The GTE-supplemented cows showed lower plasma TAG concentrations at weeks 1 and 7 and lower plasma cholesterol concentrations at weeks 4 and 7 than the control cows.

**Table 4:** Metabolic and antioxidant parameters in plasma and concentrations of lipids in the liver of cows fed the control diet (Con) or the diet supplemented with green tea extract (GTE) in weeks 1, 4 and 7 of lactation^1^

	Week 1			Week 4			Week 7			*P*-value			
	Con	GTE	SEM	Con	GTE	SEM	Con	GTE	SEM	GTE	Week	GTE × week	Parity
Plasma													
NEFA, mmol/L	0.52	0.43	0.06	0.38	0.36	0.07	0.30	0.20	0.07	0.17	< 0.001	0.48	0.66
BHBA, mmol/L	0.97	1.03	0.15	0.98	0.88	0.15	0.68	0.68	0.15	0.87	0.004	0.68	0.71
TAG, μmol/L	98.8^a^	79.6^b^	6.6	108	95.1	6.8	128^a^	99.6^b^	6.67	< 0.001	< 0.001	0.20	0.52
Cholesterol, mmol/L	2.05	1.77	0.24	3.48^a^	2.97^b^	0.24	4.97^a^	4.05^b^	0.24	0.003	< 0.001	0.007	0.017
α-Tocopherol, μmol/g lipids^2^	6.55	6.35	0.50	7.10^a^	6.08^b^	0.50	7.02	6.59	0.51	0.21	< 0.43	0.32	0.60
β-Carotene, μmol/g lipids^2^	13.5^a^	10.8^b^	1.18	10.9	9.51	1.20	13.7^a^	9.78^b^	1.18	0.007	0.036	0.29	0.05
TEAC, mmol/L	3.50	3.67	0.14	3.70	3.79	0.14	3.96	3.96	0.14	0.25	< 0.001	0.55	0.24
Liver													
TAG, μmol/g	37.1	25.5	6.3	37.2^a^	22.3^b^	6.3	15.0	9.4	6.4	0.07	< 0.001	0.24	0.57
Cholesterol, μmol/g	7.52	7.01	0.59	7.78	7.27	0.59	5.66	5.92	0.62	0.62	< 0.001	0.43	0.74

^1^Values are least square means, *n* = 15 for each group

^2^Concentrations of α-tocopherol and β-carotene are related to lipids (TAG + cholesterol)

^a,b^ Means within one week with different superscripts differ significantly (*P* < 0.05)

BHBA, β-hydroxybutyrate; NEFA, non esterified fatty acids; SEM, standard error of means; TAG, triacylglycerols; TEAC, trolox equivalent antioxidative capacity

The concentrations of α-tocopherol and β-carotene in the plasma were related to the concentrations of plasma lipids (TAG + cholesterol), as these vitamins are located within lipoproteins. The concentration of α-tocopherol was not influenced by GTE supplementation (Table 4). In contrast, the concentration of β-carotene was influenced by GTE supplementation. The GTE-supplemented cows had lower concentrations of β-carotene in the plasma at weeks 1 and 7 than the control cows. The plasma concentration of TEAC was influenced by the week of lactation but was not influenced by GTE supplementation.

### Liver TAG and cholesterol concentrations

The concentrations of TAG and cholesterol in the liver were significantly influenced by the week of lactation (Table 4). GTE supplementation caused a trend of reduction in TAG (*P* = 0.07); at week 4, the hepatic TAG concentration was significantly lower in the GTE-supplemented cows than in the control cows. The hepatic cholesterol concentration was not influenced by GTE supplementation.

### Concentrations of acute and negative acute phase proteins in the plasma

The plasma concentrations of SAA and albumin were influenced by the week of lactation (Table 5). The GTE-supplemented cows showed a reduction in the SAA plasma concentrations in weeks 1 and 4 compared to the control cows. The concentrations of HP, albumin and RBP4 did not differ between the GTE-supplemented and control cows.

**Table 5:** Concentrations of acute phase and negative acute phase proteins in plasma and relative mRNA abundances of proinflammatory genes and genes of the acute phase reaction in the liver of cows fed the control diet (Con) or the diet supplemented with green tea extract (GTE) in weeks 1, 4 and 7 of lactation^1^

	Week 1			Week 4			Week 7			*P*-value			
	Con	GTE	SEM	Con	GTE	SEM	Con	GTE	SEM	GTE	Week	GTE × week	Parity
Plasma													
SAA, μg/mL	0.33^a^	0.26^b^	0.02	0.33^a^	0.27^b^	0.02	0.29	0.25	0.02	0.007	0.029	0.52	0.41
HP, mg/mL	0.93	0.83	0.34	0.37	0.26	0.33	0.96	0.50	0.34	0.59	0.22	0.67	0.003
Albumin, g/dL	2.83	2.70	0.14	2.98	2.87	0.14	3.08	3.00	0.14	0.23	0.003	0.94	0.30
RBP4, μg/mL	1.59	1.66	0.15	1.57	1.64	0.15	1.76	1.76	0.15	0.22	0.08	0.87	< 0.001
Liver, relative mRNA abundances^2^													
*CCL2*	1.00	1.02	0.31	1.21	1.13	0.31	1.53	1.56	0.31	0.90	0.034	0.96	0.68
*IL1B*	1.00	1.20	0.18	0.86	1.05	0.18	0.94	1.00	0.18	0.65	0.33	0.78	0.018
*CXCL8*	1.00	0.96	0.36	1.36	1.93	0.36	1.74	1.66	0.36	0.48	0.001	0.20	0.61
*PTGS2*	1.00	1.10	0.22	1.18	1.28	0.22	1.01	1.22	0.22	0.27	0.38	0.90	0.44
*TNF*	1.00	1.48	0.38	1.38	1.84	0.38	1.17	1.39	0.38	0.36	0.16	0.78	0.14
*CP*	1.00	0.81	0.16	0.72	0.77	0.16	0.84	0.75	0.16	0.66	0.23	0.42	0.49
*CRP*	1.00	0.65	0.16	1.21	1.04	0.16	1.16	0.93	0.16	0.035	0.030	0.72	0.87
*HP*	1.00	0.86	0.43	0.13	0.95	0.43	0.20	0.50	0.43	0.28	0.06	0.14	0.98
*SAA3*	1.00	1.29	0.36	0.54	0.79	0.36	0.58	0.63	0.36	0.46	0.025	0.83	0.93

^1^Values are least square means, *n* = 15 for each group

^2^Relative mRNA abundances of Con in week 1 are set as a reference (=1.00)

^a,b^ Means within one week with different superscripts differ significantly (*P* < 0.05)

HP, haptoglobin; RBP4, retinol binding protein 4; SAA, serum amyloid A; SEM, standard error of means

### mRNA levels of genes related to inflammation, the acute phase reaction, the antioxidant and cytoprotective system and the unfolded protein response (UPR)

The relative mRNA abundances of inflammatory genes (*CCL2, IL1B, CXCL8, PTGS2, TNF*) in the liver were not influenced by GTE supplementation (Table 5). GTE supplementation reduced the relative mRNA abundance of CRP, an acute phase protein. In contrast, the mRNA abundances of CP, HP and SAA3, other acute phase proteins, in the liver were not influenced by GTE supplementation. Target genes of nuclear factor-E2-related factor 2 (Nrf2), which is involved in the antioxidant and cytoprotective system (*CAT, GPX1, MT1A, NQO1, SOD1, UGT1A1*), were also not influenced by GTE supplementation (Table 6). There was no significant main effect of GTE supplementation on the relative mRNA abundances of all the genes of the UPR (*ATF4, BAK1, BAX, CASP3, CASP8, DDIT3, DNAJC3, EDEM1, HERPUD1, HSPA5, PDIA4, XBP1s*) in the liver. However, at week 1 of lactation, the relative mRNA concentrations of some of the UPR genes considered (*CASP3, DNAJC3, HSPA5*) were significantly reduced in the group supplemented with GTE compared to the control group. The relative mRNA concentrations of some other genes of the UPR (*CASP8, DDIT3, EDEM1, XBP1s*) were reduced by 20% to 40% in the GTE-supplemented group; however, for these genes, the differences between the two groups were not statistically significant.

**Table 6:** Relative mRNA abundances of genes of the unfolded protein response (UPR) and genes of the Nrf2 pathway in the liver of cows fed the control diet (Con) or the diet supplemented with green tea extract (GTE) in weeks 1, 4 and 7 of lactation^1^

	Week 1			Week 4			Week 7			*P*-value			
	Con	GTE	SEM	Con	GTE	SEM	Con	GTE	SEM	GTE	Week	GTE × week	Parity
Genes of Nrf2 pathway^2^													
*CAT*	1.00	1.02	0.18	1.11	0.92	0.18	1.41	1.23	0.18	0.15	0.003	0.49	0.036
*GPX1*	1.00	1.10	0.19	0.79^a^	1.29^b^	0.19	1.02	1.21	0.19	0.25	0.67	0.12	0.053
*MT1A*	1.00	0.71	0.32	0.19	0.35	0.16	0.10	0.15	0.32	0.85	< 0.001	0.37	0.81
*NQO1*	1.00	0.90	0.17	0.97	0.84	0.17	1.09	0.97	0.17	0.19	0.38	0.98	0.34
*SOD1*	1.00	0.89	0.10	0.95	0.85	0.10	1.00	0.92	0.10	0.08	0.48	0.96	0.16
*UGT1A1*	1.00	0.98	0.17	0.88	0.67	0.17	1.04	0.92	0.17	0.17	0.07	0.64	0.34
Genes of UPR^2^													
*ATF4*	1.00	0.87	0.08	0.96	0.98	0.08	1.04	0.99	0.08	0.36	0.41	0.49	0.98
*BAK1*	1.00	1.32	0.32	0.76	1.27	0.32	0.90	1.06	0.32	0.19	0.61	0.71	0.11
*BAX*	1.00	0.94	0.19	1.49	1.43	0.19	1.12	1.25	0.19	0.71	0.001	0.71	0.12
*CASP3*	1.00^a^	0.77^b^	0.10	0.90	0.79	0.10	0.81	0.71	0.10	0.06	0.06	0.42	0.46
*CASP8*	1.00	0.76	0.14	1.07	1.13	0.14	0.97	1.01	0.14	0.82	0.017	0.10	0.28
*DDIT3*	1.00	0.73	0.14	0.97	1.15	0.14	0.96	0.91	0.14	0.96	0.051	0.028	0.08
*DNAJC3*	1.00^a^	0.57^b^	0.16	0.86	1.01	0.16	0.81	0.81	0.16	0.34	0.21	0.006	0.79
*EDEM1*	1.00	0.62	0.20	1.09	1.36	0.16	1.20	1.50	0.16	0.50	0.001	0.012	0.48
*HERPUD1*	1.00	0.89	0.16	0.69	0.77	0.16	0.76	0.76	0.16	0.79	0.10	0.66	0.48
*HSPA5*	1.00^a^	0.57^b^	0.20	0.62	0.67	0.20	0.60	0.59	0.20	0.20	0.32	0.11	0.47
*PDIA4*	1.00	0.64	0.19	0.75	0.93	0.19	0.93	0.82	0.19	0.31	0.91	0.10	0.49
*XBP1s*	1.00	0.61	0.21	0.33	0.68	0.21	0.42	0.49	0.21	0.87	0.046	0.055	0.19

^1^Values are least square means, *n* = 15 for each group

^2^Relative mRNA abundances of Con in week 1 are set as a reference (=1.00)

^a,b^ Means within one week with different superscripts differ significantly (*P* < 0.05)

*Nrf2* Nuclear factor-erythroid 2-related factor 2, *SEM* standard error of means, *UPR* unfolded protein response

### mRNA abundances of genes involved in β-oxidation, lipogenesis, VLDL assembly and ketogenesis

The relative mRNA abundance of *ACOX1* was significantly reduced by GTE supplementation (Table 7). Moreover, there was a trend of reduction in the mRNA abundance of *CPT1A* by GTE supplementation (*P* = 0.06). The relative mRNA abundances of *ACADM, SREBF1, ACACA* and *FASN* were also not influenced by GTE supplementation. GTE supplementation tended to cause a reduction in the relative mRNA abundance of *APOB* (*P* = 0.09); the relative mRNA abundance of *MTTP* was not influenced by GTE supplementation. GTE supplementation tended to cause a reduction in the relative mRNA abundance of *ACAT1* (*P* = 0.07); the relative mRNA abundances of *HMGCS2* and *HMGCL* were not influenced by GTE supplementation.

**Table 7:** Relative mRNA abundances of genes of the lipid metabolism (β-oxidation, lipogenesis, VLDL assembly) and ketogenesis in the liver of cows fed the control diet or the control diet supplemented with green tea extract (GTE) in weeks 1, 4 and 7 of lactation^1^

	Week 1			Week 4			Week 7			*P*-value			
	Con	GTE	SEM	Con	GTE	SEM	Con	GTE	SEM	GTE	Week	GTE × week	Parity
Genes of β-oxidation^2^													
*ACADM*	1.00	0.69	0.19	1.05	0.95	0.19	1.06	0.96	0.19	0.21	0.27	0.57	0.51
*ACOX1*	1.00	0.74	1.57	1.19^a^	0.84^b^	1.53	1.11	0.93	1.57	0.005	0.23	0.70	0.06
*CPT1A*	1.00^a^	0.64^b^	0.17	1.14	1.03	0.14	0.92	0.68	0.14	0.06	0.002	0.33	0.84
Genes of lipogenesis^2^													
*SREBF1*	1.00	0.85	0.44	1.49	1.37	0.44	2.22	1.33	0.44	0.13	0.018	0.34	0.56
*ACACA*	1.00	0.66	0.54	1.91	1.61	0.54	2.97	2.15	0.54	0.22	< 0.001	0.69	0.55
*FASN*	1.00	0.77	0.59	1.31	1.88	0.59	1.48	2.36	0.59	0.48	0.03	0.34	0.12
Genes of VLDL assembly^2^													
*APOB*	1.00	0.74	0.18	1.32	1.11	0.18	1.41	1.11	0.18	0.09	< 0.001	0.91	0.75
*MTTP*	1.00	0.78	0.16	1.03	0.93	0.16	0.96	0.79	0.16	0.20	0.50	0.85	0.46
Genes of ketogenesis^2^													
*ACAT1*	1.00	0.98	0.20	0.94	0.75	0.20	1.36^a^	0.84^b^	0.20	0.07	0.19	0.19	0.41
*HMGCS2*	1.00	0.74	0.27	1.09	1.29	0.27	1.21	1.45	0.27	0.96	0.031	0.28	0.30
*HMGCL*	1.00	1.08	0.22	0.79	0.80	0.22	1.01	1.02	0.22	0.89	0.11	0.94	0.09

^1^Values are least square means, *n* = 15 for each group

^2^Relative mRNA abundances of Con in week 1 are set as a reference (=1.00)

^a,b^ Means within one week with different superscripts differ significantly (*P* < 0.05)

*SEM* standard error of means, *VLDL* very low-density lipoproteins

## Discussion

In this study, cows were supplemented with GTE from 7 d before the expected day of calving until 7 d after calving. In contrast to the hypothesis of this study, supplementation with GTE did not improve milk parameters and overall had little effect on the expression of genes involved in inflammation, ER stress and lipid metabolism in the liver. The major finding of this study was that supplementation with GTE caused a reduction in the milk fat concentration. This finding contradicts the results of studies in dairy cows, in which various plant sources of polyphenols (grape seed and grape marc meal, citrus pulp, an extract of *S. baicalensis*) did not influence the fat concentration of the milk [10, 11, 28]. Based on the finding that various sources of dietary polyphenols did not adversely influence milk fat in previous studies, it is likely that the effect observed in this study is specific for green tea. Milk fat consists of three different sources of fatty acids: (I) fatty acids derived from *de novo* fatty acid synthesis in the mammary gland, (II) fatty acids derived from TAG-rich lipoproteins released by the action of lipoprotein lipase, and (III) fatty acids derived from hydrolysis of fatty acids in adipose tissue taken up into the mammary gland. From the literature, there are no indications that polyphenols from green tea affect these three sources of fatty acids. Acetate derived from rumen fermentation of carbohydrates is an important substrate for *de novo* synthesis of fatty acids in the mammary gland. It has been shown that tannins have strong effects on rumen fermentation, and they decrease the formation of acetate by the rumen microbiota [29]. However, the doses of tannins that were successful in lowering acetate production in the rumen *in vivo* are much greater than the concentration of tannic polyphenols supplied by GTE in the present study [30, 31]. Although the milk yield was not influenced by GTE supplementation, the reduction of milk fat concentration led to a reduction of the daily amount of milk fat and ECM and an improvement in the energy balance (*P* = 0.06). The cows supplemented with GTE also showed a reduced feed efficiency compared to the control cows, which might be explained by a lower energy mobilization from body stores due to the improved energy balance.

We observed that GTE supplementation tended to cause a reduction in the TAG concentration in the liver and a significant reduction in the plasma TAG concentration. Liver TAG concentration is strongly related to the energy balance, as NEFAs released from adipose tissue under a negative energy balance are the main substrates for TAG formation in the liver [32]. Therefore, it is likely that the reduced liver and plasma TAG concentrations observed in the cows supplemented with GTE might be due to low negative energy balance. The concentrations of NEFAs were approximately 20% lower in the GTE-supplemented cows than in the control cows. Although this difference was not significant, it is likely that there was a lower amount of NEFAs delivered to the liver in the GTE-supplemented cows than in the control cows. The finding that the gene expression of *ACOX1*, an enzyme involved in peroxisomal β-oxidation, was reduced and that the gene expression of *CPT1*, an enzyme involved in mitochondrial ß-oxidation, tended to show a reduction in the GTE-supplemented cows could also be due to the lower uptake of NEFAs in the liver. The expression of these two genes involved in β-oxidation of fatty acids is regulated by peroxisome proliferator-activated receptor α, a transcription factor that is activated by fatty acids [33]. We found that genes involved in lipogenesis were not influenced by GTE supplementation, suggesting that there was no effect on hepatic lipogenesis. However, lipogenesis is generally barely active in ruminant liver and thus has a negligible effect on hepatic TAG concentration in ruminants [34].

To investigate the effect of GTE on the inflammatory condition in early lactation, we determined the concentrations of two acute phase proteins, HP and SAA, in the plasma and the relative mRNA levels of a series of proinflammatory genes (*CCL2, IL1B, CXCL8, PTGS2, TNF*) and genes encoding acute phase proteins (*SAA3, CP, HP*) in the liver samples. We observed that the concentration of SAA in the plasma was reduced in the GTE-supplemented cows compared to the control cows. However, the concentration of HP as well as the relative mRNA abundances of various proinflammatory genes (*CCL2, IL1B, CXCL8, PTGES2, TNF*) and acute phase proteins (*SAA3, CP, HP*) in the liver were not different between the GTE-supplemented cows and the control cows. These findings suggest that supplementation with GTE overall had minor effects on the inflammatory condition in the dairy cows of this study.

Recently, ER stress was shown to be induced in the liver of dairy cows during early lactation [35]. To assess whether GTE supplementation attenuates the development of ER stress, we determined the relative mRNA concentrations of various genes of the UPR, an adaptive response triggered by ER stress that aims to restore ER function [36]. We observed that the relative mRNA levels of some of the genes considered (*CASP3, DNAJC3, HSPA5*) were significantly reduced in the GTE-supplemented group compared to the control group at week 1 of lactation. This finding indicates that supplementation with GTE could reduce ER stress in dairy cows during early lactation. Recently, we observed that supplementation of cows with a grape seed and grape marc meal extract lowers the expression of genes of the UPR in dairy cows [12]. Thus, the present study corroborates this recent study in suggesting that supplementation of polyphenols could be a nutritional approach to prevent the occurrence of ER stress in the liver of dairy cows during early lactation. The prevention of ER stress is relevant with respect to animal health because the UPR triggered by ER stress might contribute to the development of some of the pathophysiological events occurring in dairy cows during early lactation, such as fatty liver, ketosis or insulin resistance [37].

During the transition period, dairy cows are prone to developing oxidative stress. A high energy requirement resulting from an increased production of superoxide radicals in the mitochondrial electron chain and the existence of a proinflammatory condition associated with an increased production of free radicals by activated leucocytes are two major reasons for this finding [38]. Polyphenols are known for their strong antioxidant properties [5]. Therefore, one further aim of this study was to determine whether supplementation with GTE could improve the antioxidant system. The results of this study reveal that GTE supplementation failed to improve the antioxidant system in the first weeks of lactation. We observed that the concentration of α-tocopherol, the most powerful lipid soluble antioxidant, and the total antioxidant capacity in plasma (TEAC) remained unchanged, indicating that supplementation with GTE overall had no effect on the antioxidant system. The finding that the relative mRNA concentrations of several target genes of Nrf2, the master regulator of the antioxidant and cytoprotective system in the body, including genes encoding antioxidant enzymes such as catalase, superoxide dismutase and glutathione peroxidase, were not influenced by supplementation with GTE supports these results. The present study agrees with several other studies that showed that supplementation of plants or plant extracts rich in polyphenols does not improve the antioxidative status in dairy cows [10, 16, 39, 40].

Overall, this study showed that supplementation with GTE has only minor effects on metabolism in dairy cows. This finding could be attributed to the observation that flavonoids present in GTE are largely decomposed by ruminal microbes and thus are not available for metabolism [41]. Based on the assumption that a large part of the flavonoids from GTE was decomposed in the rumen, it is possible that the effects of GTE supplementation observed in this study, such as the reduction in milk fat content, were mainly caused by products derived from polyphenols via microbial decomposition within the rumen rather than by the native polyphenols themselves.

Our study also has some limitations that must be considered in the interpretation of the data. First, due to technical reasons, we were not able to record the feed intake in the period 5 d before the expected calving date until 5 d after calving. We cannot rule out the possibility that there were potential differences in the feed intake between the two groups during this time period, which could influence some of the variables measured in this study. Second, we used a relatively low number of cows per group with respect to the animal protection law. The selection of 15 cows per group was based on a biometric analysis for detecting a significant difference among groups for energy-corrected milk based on the effect of a recent study in which cows were fed a supplement consisting of green tea and curcuma [16]. However, we are aware that the statistical power of this study may not necessarily be sufficient to detect differences in other variables. Thus, it is possible that the potential effects of GTE on metabolic pathways, e.g., inflammation, could not be detected due to insufficient statistical power of the study. Nevertheless, our study agrees with other studies that showed that the effects of GTE flavonoids on metabolism and the antioxidant status in dairy cows might be generally limited, even if they are applied intraduodenally. Stoldt et al. [42, 43] showed that intraduodenal application of quercetin in dairy cows causes an increase in the concentration of total flavonoids in the plasma but does not influence milk, plasma concentrations of NEFA and BHBA, and several other metabolic parameters, including concentrations of glucose, cholesterol and TAG in the plasma as well as hepatic expression of genes involved in lipid metabolism and the antioxidant system.

## Conclusions

This study shows that supplementation of dairy cows with GTE in the period from 7 d before to 7 d after parturition overall has minor effects on the metabolism of dairy cows. The most obvious and relevant finding observed was a decrease in the fat concentration of the milk, an effect that cannot be explained by the data of this study. The tendency towards a reduction of the liver TAG concentration observed in the cows supplemented with GTE might be due to a slightly improved energy balance. Moreover, supplementation with GTE had minor effects on inflammation and the antioxidant system. However, there was a downregulation of some genes of the UPR at week 1 of lactation, suggesting that polyphenols could suppress the development of ER stress in dairy cows during early lactation.

## Supplementary information

**Additional file 1 Table S1.** Characteristics of gene-specific primers used for qPCR analysis in liver.

## Data Availability

The data supporting the conclusions of this article is included within the article and its additional file.

## Abbreviations

ACACA: Acetyl-CoA carboxylase
ACADM: Acyl-CoA dehydrogenase medium chain
ACAT1: Acetyl-CoA acetyl transferase 1
ACOX1: Acyl-CoA oxidase 1
ADF: Acid detergent fibre
APOB: Apolipoprotein B
ATF4: Activating transcription factor 4
BAK1: BCL2-antagonist/killer 1
BAX: BCL2 associated X, apoptosis regulator
BHBA: β-hydroxybutyrate
CASP3: Caspase 3
CASP8: Caspase 8
CAT: Catalase
CCL2: C-C motif chemokine ligand 2
CP: Crude protein
*CP*: Ceruloplasmin
CPT1A: Carnitine palmitoyl transferase 1A
CRP: C-reactive protein
CXCL8: Interleukin 8
DDIT3: DNA-damage-inducible transcript 3
DNAJC3: DnaJ heat shock protein family (Hsp40) member C3
DMI: Dry matter intake
EC: Energy-corrected milk
EDEM1: ER degradation enhancing alpha-mannosidase like protein 1
EEF1A1: Eukaryotic translation elongation factor 1 alpha 1
ER: Endoplasmic reticulum
FASN: Fatty acid synthase
GPX1: Glutathion peroxidase 1
H3F3A: H3 histone familiy member 3A
HERPUD1: Homocysteine inducible ER protein with ubiquitin like domain 1
GTE: Green tea extract
HP: Haptoglobin
IL1B: Interleukin 1 beta
HMGCL: 3-Hydroxymethyl-3-methylglutaryl-CoA lyase
HMGCS2: 3-Hydroxy-3-methylglutaryl-CoA synthase 2
HSPA5: Heat shock protein family A (Hsp70) member 5
MT1A: Metallothionein-1A
MTTP: Microsomal triglyceride transfer protein
NDF: Neutral detergent fibre
NEL: Net energy lactation
Nrf2: nuclear factor E2 related factor 2
NQO1: NAD(P)H-quinon oxidoreductase
NEFA: Non esterified fatty acids
PDIA4: Protein disulfide isomerase family A member 4
PTGS2: Prostaglandin endoperoxide synthase 2
RBP4: Retinol-binding protein 4
RPL12: Ribosomal protein L12
SAA: Serum amyloid A
SAA3: Serum amyloid A3
SOD1: Superoxide dismutase 1
SREBF1: Sterol regulatory element-binding transcription factor 1
TAG: Triacylglycerols
TEAC: Trolox equivalent antioxidant capacity
TMR: Total mixed ration
TNF: Tumor necrosis factor
UGT1A1: UDP-glucuronosyl transferase family 1 member A1
UPR: Unfolded protein response
XBP1s: Spliced X-box binding protein 1

## References

[1] Bertoni G, Trevisi E, Han X, Bionaz M (2008). Effects of inflammatory conditions on liver activity in puerperium period and consequences for performance in dairy cows. J Dairy Sci.

[2] Seo J, Osorio JS, Schmitt E, Corrêa MN, Bertoni G, Trevisi E (2014). Hepatic purinergic signaling gene network expression and its relationship with inflammation and oxidative stress biomarkers in blood from peripartal dairy cattle. J Dairy Sci.

[3] Du X, Liu G, Loor JJ, Fang Z, Bucktrout R, Yang Y (2018). Impaired hepatic autophagic activity in dairy cows with severe fatty liver is associated with inflammation and reduced liver function. J Dairy Sci.

[4] Bradford BJ, Yuan K, Farney JK, Mamedova LK, Carpenter AJ (2015). Invited review: inflammation during the transition to lactation: new adventures with an old flame. J Dairy Sci.

[5] Gessner DK, Ringseis R, Eder K (2017). Potential of plant polyphenols to combat oxidative stress and inflammatory processes in farm animals. J Anim Physiol Anim Nutr (Berl).

[6] Trevisi E, Amadori M, Cogrossi S, Razzuoli E, Bertoni G (2012). Metabolic stress and inflammatory response in high-yielding, periparturient dairy cows. Res Vet Sci.

[7] Bossaert P, Trevisi E, Opsomer G, Bertoni G, De Vliegher S, Leroy JL (2012). The association between indicators of inflammation and liver variables during the transition period in high-yielding dairy cows: an observational study. Vet J.

[8] Carpenter AJ, Ylioja CM, Vargas CF, Mamedova LK, Mendonça LG, Coetzee JF (2016). Hot topic: Early postpartum treatment of commercial dairy cows with nonsteroidal antiinflammatory drugs increases whole-lactation milk yield. J Dairy Sci.

[9] Trevisi E, Bertoni G, Quinn PI (2008). Attenuation with acetylsalicylate treatments of inflammatory conditions in periparturient dairy cows. Aspirin and health research progress.

[10] Gessner DK, Koch C, Romberg FJ, Winkler A, Dusel G, Herzog E (2015). The effect of grape seed and grape marc meal extract on milk performance and the expression of genes of endoplasmic reticulum stress and inflammation in the liver of dairy cows in early lactation. J Dairy Sci.

[11] Olagaray KE, Brouk MJ, Mamedova LK, Sivinski SE, Liu H, Robert F (2019). Dietary supplementation of Scutellaria baicalensis extract during early lactation decreases milk somatic cells and increases whole lactation milk yield in dairy cattle. PLoS One.

[12] Gessner DK, Winkler A, Koch C, Dusel G, Liebisch G, Ringseis R (2017). Analysis of hepatic transcript profile and plasma lipid profile in early lactating dairy cows fed grape seed and grape marc meal extract. BMC Genomics.

[13] Khan Naghma, Mukhtar Hasan (2018). Tea Polyphenols in Promotion of Human Health. Nutrients.

[14] Wang L, Yang G, Yuan L, Yang Y, Zhao H, Ho CT (2019). Green Tea Catechins Effectively Altered Hepatic Fibrogenesis in Rats by Inhibiting ERK and Smad1/2 Phosphorylation. J Agric Food Chem.

[15] Xia HM, Wang J, Xie XJ, Xu LJ, Tang SQ (2019). Green tea polyphenols attenuate hepatic steatosis, and reduce insulin resistance and inflammation in high-fat diet-induced rats. Int J Mol Med.

[16] Winkler A, Gessner DK, Koch C, Romberg FJ, Dusel G, Herzog E (2015). Effects of a plant product consisting of green tea and curcuma extract on milk production and the expression of hepatic genes involved in endoplasmic stress response and inflammation in dairy cows. Arch Anim Nutr.

[17] Singleton-VL, Rossi J (1965). Colorimetry of total phenolics with phosphomolybdic-phosphotungstic acid reagents. Am J Enol Vitic.

[18] GfE (Gesellschaft für Ernährungsphysiologie), editor. Empfehlungen zur Energie- und Nährstoffversorgung der Milchkühe und Aufzuchtrinder. Frankfurt am Main: DLG-Verlag; 2001.

[19] VDLUFA (Verband Deutscher Landwirtschaftlicher Untersuchungs- und Forschungsanstalten), editor. Handbuch der Landwirtschaftlichen Versuchs-und Untersuchungsmethodik (VDLUFA-Methodenbuch), Bd. III. Die chemische Untersuchung von Futtermitteln. Darmstadt: VDLUFA-Verlag; 2006.

[20] Van Soest PJ, Robertson JB, Lewis BA. Methods for dietary fiber, neutral detergent fiber, and nonstarch polysaccharides in relation to animal nutrition. J Dairy Sci. 1991;74:3583–97. 10.3168/jds.S0022-0302(91)78551-2 1660498

[21] Re R, Pellegrini N, Proteggente A, Pannala A, Yang M, Rice-Evans C (1999). Antioxidant activity applying an improved ABTS radical cation decolorization assay. Free Radic Biol Med.

[22] Balz MK, Schulte E, Their HP (1993). 1993. Simultaneous determination of tocopheryl acetate, tocopherols and tocotrienols by HPLC with fluorescence detection in foods. Fat Sci Technol.

[23] Hara A, Radin NS (1978). Lipid extraction of tissues with a low-toxicity solvent. Anal Biochem.

[24] Eder K, Kirchgessner M (1994). Dietary fat influences the effect of zinc deficiency on liver lipids and fatty acids in rats force-fed equal quantities of diet. J Nut.

[25] Gessner DK, Fiesel A, Most E, Dinges J, Wen G, Ringseis R (2013). Supplementation of a grape seed and grape marc meal extract decreases activities of the oxidative stress-responsive transcription factors NF-kappaB and Nrf2 in the duodenal mucosa of pigs. Acta Vet Scand.

[26] Gessner DK, Schlegel G, Keller J, Schwarz FJ, Ringseis R, Eder K (2013). Expression of target genes of nuclear factor E2-related factor 2 in the liver of dairy cows in the transition period and at different stages of lactation. J Dairy Sci.

[27] Vandesompele Jo, De Preter Katleen, Pattyn Filip, Poppe Bruce, Van Roy Nadine, De Paepe Anne, Speleman Frank (2002). Genome Biology.

[28] de Lima LS, Santos GT, Schogor AL, de Marchi FE, de Souza MR, Santos NW (2015). Effect of abomasal or ruminal administration of citrus pulp and soybean oil on milk fatty acid profile and antioxidant properties. J Dairy Res.

[29] Vasta V, Daghio M, Cappucci A, Buccioni A, Serra A, Viti C (2019). Invited review: Plant polyphenols and rumen microbiota responsible for fatty acid biohydrogenation, fiber digestion, and methane emission: Experimental evidence and methodological approaches. J Dairy Sci.

[30] Moate PJ, Williams SR, Torok VA, Hannah MC, Ribaux BE, Tavendale MH (2014). Grape marc reduces methane emissions when fed to dairy cows. J Dairy Sci.

[31] Rira M, Morgavi DP, Archimède H, Marie-Magdeleine C, Popova M, Bousseboua H (2015). Potential of tannin-rich plants for modulating ruminal microbes and ruminal fermentation in sheep. J Anim Sci.

[32] Bobe G, Young JW, Beitz DC (2004). Invited review: pathology, etiology, prevention, and treatment of fatty liver in dairy cows. J Dairy Sci.

[33] Desvergne B, Wahli W (1999). Peroxisome proliferator-activated receptors: nuclear control of metabolism. Endocr Rev.

[34] Ballard FJ, Hanson RW, Kronfeld DS (1969). Gluconeogenesis and lipogenesis in tissue from ruminant and nonruminant animals. Fed Proc.

[35] Gessner DK, Schlegel G, Ringseis R, Schwarz FJ, Eder K (2014). Up-regulation of endoplasmic reticulum stress induced genes of the unfolded protein response in the liver of periparturient dairy cows. BMC Vet Res.

[36] Cnop M, Foufelle F, Velloso LA (2012). Endoplasmic reticulum stress, obesity and diabetes. Trends Mol Med.

[37] Ringseis R, Gessner DK, Eder K (2015). Molecular insights into the mechanisms of liver-associated diseases in early-lactating dairy cows: hypothetical role of endoplasmic reticulum stress. J Anim Physiol Anim Nutr (Berl).

[38] Abuelo A, Hernández J, Benedito JL, Castillo C (2019). Redox Biology in Transition Periods of Dairy Cattle: Role in the Health of Periparturient and Neonatal Animals. Antioxidants (Basel).

[39] Gobert M, Martin B, Ferlay A, Chilliard Y, Graulet B, Pradel P (2009). Plant polyphenols associated with vitamin E can reduce plasma lipoperoxidation in dairy cows given n-3 polyunsaturated fatty acids. J Dairy Sci.

[40] Gohlke A, Ingelmann CJ, Nürnberg G, Weitzel JM, Hammon HM, Görs S (2013). Influence of 4-week intraduodenal supplementation of quercetin on performance, glucose metabolism, and mRNA abundance of genes related to glucose metabolism and antioxidative status in dairy cows. J Dairy Sci.

[41] Wein S, Beyer B, Gohlke A, Blank R, Metges CC, Wolffram S (2016). Systemic absorption of Catechins after Intraruminal or Intraduodenal application of a green tea extract in cows. PLoS One.

[42] Stoldt AK, Derno M, Nürnberg G, Weitzel JM, Otten W, Starke A (2015). Effects of a 6-wk intraduodenal supplementation with quercetin on energy metabolism and indicators of liver damage in periparturient dairy cows. J Dairy Sci.

[43] Stoldt AK, Mielenz M, Nürnberg G, Sauerwein H, Esatbeyoglu T, Wagner AE (2016). Effects of a six-week intraduodenal supplementation with quercetin on liver lipid metabolism and oxidative stress in peripartal dairy cows. J Anim Sci.

