# Supplementary material for: Effects of supplementation of green tea extract on the milk performance of peripartal dairy cows and the expression of stress response genes in the liver
Source: J Anim Sci Biotechnol. 2020 Jun 5;11:57. doi: 10.1186/s40104-020-00465-y (PMC7273663; doi:10.1186/s40104-020-00465-y)
Supplement: Supplementary file 1 — Additional file 1 Table S1. Characteristics of gene-specific primers used for qPCR analysis in liver. [file 40104_2020_465_MOESM1_ESM.docx]

**Table S1**

Characteristics of gene-specific primers used for qPCR analysis in liver.

| Gene | | | Forward (5´ to 3´)  Reverse (5´to 3´) | | | | Annealing temperature, °C | | PCR product size, bp | | NCBI GenBank accession no. | Slope | *R^2^* | E |
| --- | --- | --- | --- | --- | --- | --- | --- | --- | --- | --- | --- | --- | --- | --- |
| Reference genes | | | | | | |  | |  | |  |  |  |  |
| *EEF1A1* | | | GTCAAAGATGTCCGTCCGTGGC  TGGCAGCGTCACCAGATTTCA | | | | 60 | | 253 | | NM_174535.2 | 1.95 | -3.35 | 1.00 |
| *H3F3A* | | | CTGTGGCACTCCGTGAAATTA  ACTTGCCTCCTGCAAAGCAC | | | | 60 | | 154 | | NM_001014389.2 | 1.94 | -3.48 | 1.00 |
| *RPL12* | | | GGCAACTGGTGATTGGAAGG  TCTGCTTCTTTCTGTCCCTTGG | | | | 60 | | 143 | | NM_205797.1 | 1.90 | -3.59 | 1.00 |
| Target genes | | | | | | |  | |  | |  |  |  |  |
| *ACACA* | | CTCTTCCGACAGGTTCAAGC  AGTCCCCGCACTCACATAAC | | | | | 60 | | 270 | | NM_174224 | 2.03 | -3.26 | 1.00 |
| *ACADM* | | GCGAGTACCCTGTCCCATTA  CCTCAGTCATTCTCCCCAAA | | | | | 57 | | 243 | | NM_001075235.1 | 1.92 | -3.53 | 0.99 |
| *ACAT1* | | TGCGCCTGCATATGCTGTTCCT  AGCACCTCCTCCTCCGTTGC | | | | | 62 | | 277 | | NM_001046075.1 | 1.94 | -3.47 | 1.00 |
| *ACOX1* | | TCAGCCGTAGCCAGCGTTAC  GTCTGGGCGTAGGTGCCAAT | | | | | 60 | | 256 | | NM_001035289.3 | 1.92 | -3.53 | 0.99 |
| *APOB* | | CGACCATCACCGTGCCCGAG  ACAAGGGCGAGGCCACCTCA | | | | | 60 | | 123 | | XM_010817552.1 | 1.97 | -3.40 | 0.98 |
| *ATF4* | | \| TGGTCTCAGACAACAGCAAG \| \| --- \| \| AGCTCATCTGGCATGGTTTC \| | | | | | 59 | | 130 | | NM_001034342.2 | 1.95 | -3.46 | 0.98 |
| *BAK1* | | TACTTCACCAAGATCGCGTC  ACGATGGCTACGCTCTTGAT | | | | | 59 | | 254 | | NM_001077918.1 | 1.97 | -3.39 | 0.98 |
| *BAX* | | TCTGACGGCAACTTCAACTG  ATGGTCACTGTCTGCCATGT | | | | | 59 | | 224 | | NM_173894.1 | 1.90 | -3.58 | 1.00 |
| *CASP3* | | AGCACCTGGTTACTTTTCCTGG  CCGAGTAAGAATGTGCATGAGC | | | | | 59 | | 118 | | NM_001077840.1 | 2.02 | -3.27 | 0.99 |
| *CASP8* | | TACCAGCGAGGAGGAGATGAAG  CATCCAGCTTACATTTGGCAATC | | | | | 59 | | 164 | | NM_001045970.2 | 1.91 | -3.55 | 0.99 |
| *CAT* | | TGGGACCCAACTATCTCCAG  AAGTGGGTCCTGTGTTCCAG | | | | | 59 | | 178 | | NM_001035386.2 | 1.92 | -3.52 | 1.00 |
| *CCL2* | | TCACAGTAGCTGCCTTCAGCA  ACCCATTTCTGCTTGGGGTCT | | | | | 60 | | 214 | | NM_174006.2 | 1.91 | -3.55 | 1.00 |
| *CP* | | CGGTGGAGGTGGAATGGGAT  TGTGCTGTCAGTGAATTGCCG | | | | | 59 | | 161 | | NM_001256556 | 1.96 | -3.42 | 0.99 |
| *CPT1A* | | CGTCGCTCGCTCACTCGG  CGGAGGTCGATCCCATCTGGA | | | | | 60 | | 137 | | NM_001304989.2 | 2.07 | -3.16 | 0.98 |
| *CRP* | | GGCCAGACAGACTTGCATAAGAAGG  GGGTTCGGGCCAGCTCTGTG | | | | | 60 | | 142 | | NM_001144097.1 | 1.96 | -3.43 | 1.00 |
| *DDIT3* | | AGTCACTGCCTTTCTCCTTC  TCTTCCTCCTTGTTTCCAGG | | | | | 59 | | 133 | | NM_001078163.1 | 1.92 | -3.53 | 0.99 |
| *DNAJC3* | | GTACGAAGGTGCTGAATGTG  ATCAGGGTCACCATCTACTG | | | | | 59 | | 133 | | NM_174756.3 | 1.92 | -3.54 | 0.99 |
| *EDEM1* | | CCCCTACCCTCGGGTGAATCT  GTGGAATCCCCCAGCAGTCG | | | | | 60 | | 126 | | NM_001103092.2 | 1.91 | -3.54 | 0.99 |
| *FASN* | | GCTGAGCCTGATGCGTCTGAGC  GGATGGCAGTGAGGCTCACGAA | | | | | 64 | | 138 | | NM_001012669.1 | 1.96 | -3.41 | 0.99 |
| *GPX1* | | GCTCTGGATTCGGAAACGGA  GAGGACAGGTTGAAGGGCTC | | | | | 59 | | 134 | | NM_174076.3 | 1.89 | -3.61 | 1.00 |
| *HERPUD1* | | CCGTGTTTCTCAGTATCCTC  TCTTGATTCACAGCCTCCTG | | | | | 59 | | 169 | | NM_001102265.2 | 1.96 | -3.42 | 0.99 |
| *HMGCL* | | AGAAGCCACCAGCTTCGTGT  GCGACTTTAGCCGGGGAGAT | | | | | 60 | | 345 | | NM_001075132.1 | 1.89 | -3.61 | 1.00 |
| *HMGCS2* | | GCCCAATATGTGGACCAAAC  ATGGTCTCAGTGCCCACTTC | | | | | 60 | | 209 | | NM_001045883.1 | 1.91 | -3.56 | 1.00 |
| *HP* | | TGAGGCAGTGTGCGGGAAGCC  AGCGTGGCTCCCGAGATGAGGTT | | | | | 60 | | 138 | | NM_001040470.2 | 1.93 | -3.51 | 0.98 |
| *HSPA5* | | CAAGTTGATGTTGGAGGTGG  AAGCCTCAGCAGTTTCCTTC | | | | | 59 | | 94 | | NM_001075148.1 | 1.89 | -3.62 | 0.98 |
| *IL1B* | | GCAGCTGGAGGAAGTAGACCCC  TGTCCCAGGAAGACGGGCCTTT | | | | | 60 | | 168 | | NM_174093.1 | 2.09 | -3.12 | 1.00 |
| *CXCL8* | | CTGCAGTTCTGTCAAGAATGA  CTCTTCACAAATACCTGCACA | | | | | 56 | | 220 | | NM_173925.2 | 1.99 | -3.35 | 0.99 |
| *MT1A* | | ATCCGACCAGTGGATCTGCTTTGCC  AGACACAGCCCTGGGCACACT | | | | | 63 | | 209 | | NM_001040492.2 | 1.98 | -3.37 | 0.99 |
| *MTTP* | | GCACCGAGAGCGTTATCCTCCA  GGAGTGTGGTGGCGGCAAGG | | | | | 64 | | 378 | | NM_001101834.1 | 1.96 | -3.42 | 1.00 |
| *NQO1* | | GGTGCTCATAGGGGAGTACGC  CGGGAGTGTGCCCAATGCTAT | | | | | 60 | | 236 | | NM_001034535.1 | 1.90 | -3.59 | 0.99 |
| *PDIA4* | | AGGTTTGACGTGAGTGGCTA  CATCGAAGTTGTCCTTGGTC | | | | | 59 | | 175 | | NM_001045879.2 | 2.04 | -3.23 | 0.99 |
| *PTGS2* | | CGCACCTCACCAAAACGGTC  ACTGCTAGGCTTCTACAGTTCGG | | | | | 60 | | 116 | | NM_174445.2 | 1.92 | -3.53 | 1.00 |
| *SAA3* | | CCTCAAGGAAGCTGGTCAAGGGGCT  CAGGCACCCCCAGGTCCCCT | | | | | 61 | | 141 | | NM_181016.3 | 1.94 | -3.47 | 0.99 |
| *SOD1* | | TGTTGCCATCGTGGATATTG  CAGCGTTGCCAGTCTTTGTA | | | | | 56 | | 143 | | NM_174615.2 | 1.92 | -3.54 | 1.00 |
| *SREBF1* | | AACGCCATCGAGAAACGCTA  CTTTTGTGGACAGCAGTGCG | | | | | 59 | | 197 | | NM_001113302.1 | 1.91 | -3.55 | 1.00 |
| *TNF* | | GCCCCCAGGGCTCCAGAAGTT  GCCACCTGGGGACTGCTCTTCC | | | | | 64 | | 134 | | NM_173966.3 | 1.91 | -3.56 | 0.98 |
| *UGT1A1* | | GCTCGTCAAGTGGCTGCCCCA  TCCCCGGGTCTCCATGCGCT | | | | | 61 | | 175 | | NM_001105636.1 | 1.89 | -3.63 | 1.00 |
| *XBP1s* | | GCTGAGTCCGCAGCAGGTG  AGGGATGGAGAAGGGGAGGC | | | | | 61 | | 236 | | NM_001271737.1 | 2.08 | -3.15 | 0.99 |
|  |  | | |  |  |  | |  | |  |  |  |  |  |
